# 573. Creation of a Statewide Ambulatory Urinary Antibiogram and Evaluation of Regional and Temporal Variation in Antibiotic Susceptibility

**DOI:** 10.1093/ofid/ofad500.642

**Published:** 2023-11-27

**Authors:** Dusica Curanovic, Jacinta Chin, Chris Garcia, Catherine Wright, Rita Stainback, Melissa Martin, Samia Naccache, Kristen Smith, Howard Engler, Kelly Flett

**Affiliations:** Labcorp, Bethesda, Maryland; Novant Health, Charlotte, North Carolina; Mayo Clinic, Rochester, Minnesota; Labcorp, Bethesda, Maryland; Labcorp, Bethesda, Maryland; Labcorp, Bethesda, Maryland; Labcorp, Bethesda, Maryland; bioMerieux, Burlington, North Carolina; Labcorp, Bethesda, Maryland; Novant Health, Charlotte, North Carolina

## Abstract

**Background:**

Empiric antibiotic selection in outpatient care often relies on inpatient antibiograms which may not accurately reflect ambulatory susceptibility rates. To address this, Novant Health (NH) and Labcorp analyzed urine culture results from North Carolina (NC) to create an ambulatory antibiogram and assess temporal and regional differences.

**Methods:**

Cross-sectional study of urine culture data collected 1/1/20-12/31/21 from ambulatory sites in NC. Testing and antibiogram preparation were performed at Labcorp (Burlington, NC) based on CLSI guidelines (M100 S28). Pediatric and adult data were compiled separately. NH and state-wide data were analyzed using the chi-square or Fisher exact test with Bonferroni correction for multiple comparisons.

**Results:**

In 2021, *Escherichia coli* was the most common isolate for NH (n=18937 adult, n=624 pediatric) and state-wide (n=87,226 adult; n=2696 pediatric). *E. coli* was ≥ 80% susceptible to all tested antibiotics except ampicillin; *Klebsiella pneumoniae* was ≥ 80% susceptible to all except nitrofurantoin; and *Proteus mirabilis*/*penneri* was ≥ 80% susceptible to all antibiotics tested. Susceptibility rates were similar between NH and state-wide data except for lower susceptibility to ceftriaxone (94% vs 95%; p< .001). Compared to 2020, state-wide adult *E. coli* isolates from 2021 had increased susceptibility for amoxicillin/clavulanic acid (86% vs 85%), ampicillin (60% vs 59%), cephalosporins (84-93% vs 82-92%), and quinolones (87% vs 86%) (p< .001 all). NH isolates did not show significant changes. Susceptibility rates by region were similar, except for lower susceptibility to cefuroxime among adult *E. coli* isolates in the Triad region relative to state-wide (82% vs 84%; p< .001). No significant temporal or regional differences in susceptibility were observed for *K. pneumoniae*, *Proteus* spp., *P. aeruginosa*, and among pediatric samples.

**Conclusion:**

Susceptibility data were largely similar in NH vs. state-wide data, and between regions. Small but statistically significant temporal changes were found in state-wide data. Overall, our results support use of a state-wide antibiogram to inform treatment choices across all regions and within specific healthcare systems.

**Disclosures:**

**Dusica Curanovic, PhD**, Labcorp: employed by labcorp during study|Labcorp: Stocks/Bonds **Chris Garcia, MD**, Labcorp: Prior employee|Labcorp: Stocks/Bonds **Catherine Wright, PhD**, Labcorp: Employee|Labcorp: Stocks/Bonds **Rita Stainback, MS, MT (ASCP) SM, DLM**, Labcorp: Employee|Labcorp: Stocks/Bonds **Melissa Martin, BS**, Labcorp: Employee **Samia Naccache, PhD, D(ABMM)**, Labcorp: Employee|Labcorp: Stocks/Bonds **Kristen Smith, PhD**, bioMerieux: Employee|Labcorp: Stocks/Bonds **Howard Engler, PhD, D(ABMM)**, Labcorp: Employee|Labcorp: Stocks/Bonds

